# Evaluation of a Nanodispersion Formulation Prepared through Microfluidic Reactors for Pulmonary Delivery of Budesonide Using Nebulizers 

**Published:** 2014

**Authors:** Hany SM Ali, Peter York, Amir Amani, Nicholas Blagden

**Affiliations:** a *Institute of Pharmaceutical Innovation, University of Bradford, Bradford, BD7 1DP, UK. *; b *Department of Pharmaceutics, Faculty of Pharmacy, Assiut University, Egypt.*; c *Department of Medical Nanotechnology, School of Advanced Technologies in Medicine, Tehran University of Medical Sciences, Tehran, Iran. *; d *Medical Biomaterials Research Center, Tehran University of Medical Sciences, Tehran, Iran. *; e *School of Pharmacy, College of Science, University of Lincoln, LN6 7TS, UK. *

**Keywords:** Microreactors, Budesonide, Nanodispersion, Aerosolization, Pulmonary delivery, Nebulizer

## Abstract

This study aimed to determine the aerosolization behavior of a nanodispersion of budesonide, prepared using microfluidic reactors. The size and morphology of budesonide nanoparticles were characterized by photon correlation spectroscopy (PCS) and transmission electron microscopy (TEM). Processing/formulation parameters for formation of the nanoparticles were studied to determine their effects on the particle size. Results showed a narrow distribution for budesonide nanodispersion with spherical and smooth surfaced particles. To investigate the *in-vitro *aerosolization performance of the nanodispersion, the preparation was compared with a commercially available budesonide microsuspension using the Comité Européen Normalization (CEN) methodology. Aerosolization results showed that the fine particle fraction (FPF) generated from the budesonide nanodispersion was significantly higher than that of the marketed budesonide (*ie*. mean (SD) 56.88 (3.37)% vs. 38.04 (7.82)%, respectively). Additionally, mass median aerodynamic diameter (MMAD) of nano-budesonide dispersion was significantly smaller than the microsuspension (*ie*. mean (SD) 3.91 (0.49) vs. 6.22 (1.09) μm, respectively), with nebulization time of nano-budesonide dispersion significantly shorter than the marketed budesonide microsuspension (*ie*. 12.3 (0.37) vs. 14.85 (0.36) min, respectively). The produced nanodispersion was found to be stable over a period of 10 days if stored at 4 °C.

## Introduction

Nanosizing, or reduction of particle size down to the sub-micron levels (1), has been widely used in recent years to improve several properties of pharmaceuticals *e.g*. their bioavailability (2, 3). Typically, nanosizing can be achieved through top-down (breaking of larger drug particles into smaller ones) or bottom-up (building of nanoparticles from molecular scale components) approaches. A recently popularized approach in bottom-up methods is precipitation using microfluidic reactors. Microfluidic reactors comprise microchannel having laminar fluids, meaning that adjacent streams of miscible fluids flow through microchannels side by side, with minimum turbulence (4). Fluid mixing in such reactors results from diffusion of molecules across the interface between fluid streams (5). Such a unique laminar flow can be exploited to generate nano-sized drug particles through controlled antisolvent precipitation in microreactors (6-8).

Administration of marketed budesonide suspension (*e.g. *Pulmicort® Respules®) by nebuilization is common in pediatric patients and adults who show difficulties in coordination of breathing when using DPIs and/or lack sufficient inspiratory capabilities (9, 10). However, several limitations are associated with the use of marketed microsuspensions of budesonide. Unintended deposition and possible low bioavailability of the drug, insufficient interactions with respiratory surfaces, lengthy treatment time and problems in administration with faster nebulizers have been reported with nebulized microsuspensions (11). Moreover, the unwanted deposition may cause localized immune suppression and local side effects such as oral yeast infections as well as the increased risk of systemic absorption (12). 

Although several studies on nebulization of nanodispersions have been reported (13), works on nebulization of nanodispersions of budesonide are limited. Jacob and Müller have reported improved respirable fraction of budesonide prepared using high pressure homogenizer in a jet nebulizer with particle size ~ 500-600 nm (14). In a clinical study, similar pulmonary absorption was reported for nano and micro suspension with a faster delivery rate in nanosuspension (9). An *in-vitro *study showed the potential of a submicron suspension of budesonide to deliver substantially more fine particles compared with the microsuspension (15). A clinical study of the submicron formulation showed no side effect compared with the marketed formulation. The improved absorption and drug delivery rate was also reported in this study (12). Nevertheless, considering the mentioned studies, no comprehensive work so far has reported the aerodynamic behavior of the nebulized nanodispersion of budesonide. Parameters such as fine particle fraction (FPF), mass median aerodynamic diameter (MMAD), geometric standard deviation (GSD) and emitted dose must be addressed for any proposed preparation as a part of documentation process. However, such parameters have not been detailed for nanodispersions of budesoinde, especially in case of preparations with particles sizes < 200nm where nanosizing shows its real effects such as substantial increases in solubility (16, 17). The aim of this study was to formulate and evaluate a nano-budesonide formulation suitable for nebulization and its aerodynamic characteristics compared to the commercially available formulation. 

## Experimental

*Materials*

Micronized budesonide (Pharm. grade), polysorbate 80 and sodium chloride (Pharm grade) were purchased from Industriale Chimica s.r.l. (Italy) and Sigma-Aldrich (Germany), respectively. Absolute ethanol, acetonitrile and methanol (HPLC grade) were from Fisher Scientific *Ltd *(UK). Potassium dihydrogen orthophosphate and disodium hydrogen phosphate were purchased from VWR (UK). Pulmicort Respules® 1 mg/2mL was from AstraZeneca (UK). Distilled water was obtained using Purelab^TM^ (ELGA, UK). 

*Solubility study*

Considering the safety concerns, to prepare a formulation for pharmaceutical purposes, for the process of budesonide nanoprecipitation, ethanol and water were chosen as solvent and antisolvent, respectively. Accordingly, solubility of budesonide in ethanol, water and different combinations of mixture of ethanol-water was checked. Excess amounts of budesonide were added to sealed vials containing ethanol, water or mixtures. All dispersions were shaken for 24 h at room temperature (25 °C). The dispersions were then filtered using hydrophilic Durapore filters (0.45 μm, Milipore, Ireland). To determine the amount of drug dissolved, UV spectroscopy was used employing V-530 UV-Vis spectrophotometer (Jasco, Japan) at wavelength 240 nm. Aliquots were examined and the solubility of drug was identified in each sample. Experiments were carried out in triplicates.

*Preparation of budesonide nanodispersions*

Budesonide nanodispersions (ND) were prepared using configured microfluidic reactors (Internal diameter 0.5 and 1 mm, fluid inlet angle 10, 25 and 50°) as detailed previously (7). The effect of different factors possibly affecting the nanoprecipitation process, namely, antisolvent flow rate, drug concentration, internal diameter, and inlet angles of microreactor was also examined. 

The inhalation studies employed the budesonide nanodispersion formulation with the smallest particle size in Table 1 (*i.e*. sample No. 5). To equalize the concentration of budesonide in the sample understudy and that of commercial suspension (*i.e*. Pulmicort Respules®, 0.5 mg/mL), the following approach was used: before nebulization studies, the preparation was further diluted with a solution of sodium chloride and polysorbate 80 to obtain a final concentration of 0.5 mg/mL, 0.9 %(W/V) and 0.02 %(W/V) for budesonide, sodium chloride and polysorbate 80, respectively (budesonide ND).

**Table 1:** The impact of experimental conditions and microreactor set up on budesonide mean particles size (n=3).

**Sample No.**	**Input variables**					**Output variable**
	**Antisolvent flow rate (mL/min)**	**Solvent flow rate (mL/min)**	**Drug concentration in the solvent (mg/mL)**	**Internal diameter (mm)**	**Inlet angle (°)**	**Mean (SD) particle size (nm)**
1	0.5	0.5	5	1.0	10	258 (2.3)
2	1.0	0.5	5	1.0	10	236 (7.5)
3	1.5	0.5	5	1.0	10	212 (3.3)
4	2.0	0.5	5	1.0	10	172 (3.2)
5	2.5	0.5	5	1.0	10	160 (4.0)
6	0.5	0.5	10	1.0	10	242 (4.8)
7	0.5	0.5	15	1.0	10	231 (5.4)
8	0.5	0.5	20	1.0	10	225 (6.2)
9	0.5	0.5	5	0.5	10	225 (6.2)
10	0.5	0.5	5	0.5	25	276 (8.2)
11	0.5	0.5	5	0.5	50	303 (7.3)

*Particle size measurement*

The average particle size diameter (Z-Ave) and the polydispersity index (PDI) of the samples were determined by photon correlation spectroscopy (PCS) technique using Zetasizer® NanoS (Malvern Instruments, UK). Dispersions were analyzed without dilution and the mean Z-Ave and polydispersity index (PDI) of three measurements was recorded. The accuracy of the instrument was calibrated by a Nanosphere^TM ^size standard, 500 nm (Duke Scientific Corporation, USA).

*Measurement of zeta potential*

Zeta potential of the budesonide ND was measured by a Zetasizer® NanoS (Malvern Instruments, UK). The measurement was performed without dilution. All measurements were made in triplicate and the mean values were reported.

*Morphology of budesonide particles*

The morphology of different budesonide particles was examined using transmission electron microscopy (TEM) or scanning electron microscopy (SEM). For budesonide ND, drops of the dispersion were placed on a carbon grid, stained with 2% uranyl acetate solution and transferred to the TEM (JEM-1200EX, Japan Electron Optics Laboratory Corporation, Japan) operated at 120 kV. Unprocessed budesonide and budesonide of the commercial suspension (adsorbed on carbon grid and air-dried) were examined by SEM (Quanta 400, FEI Company, Cambridge, UK) after being mounted onto a graphite layer on an aluminum cylinder under vacuum.

*Aerosolization*

*Aerosol output rate*

**Figure 1:**
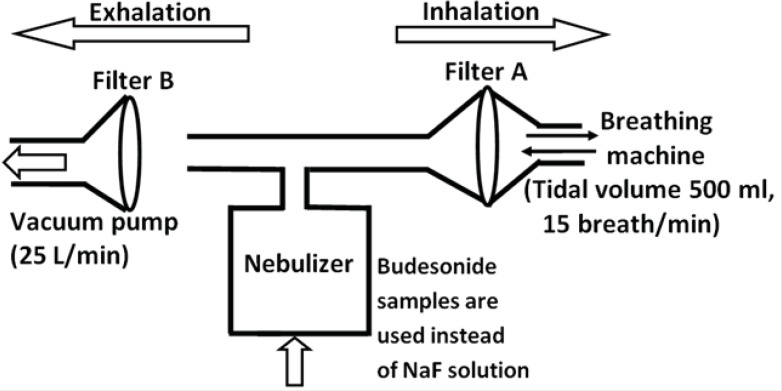
Schematic diagram of aerosol output system

The aerosol output of the tested preparations (budesonide ND and the commercial budesonide) was determined according to the Comité Européen de Normalisation (CEN) methodology (see Figure 1) (18). As discussed previously (19), the amount of budesonide was determined directly instead of a using fluoride tracer. 4 mL of each sample was introduced into the nebulizer chamber of the Sidestream jet nebulizer (the Respironics, UK). As shown in Figure 1, a breathing simulation machine (Pari GmbH, Germany) was set at a sinus flow of 15 breaths per minute with an inhalation: exhalation ratio of 1:1 and a tidal volume of 500 mL. Two electrostatic filters (Pari GmbH, Germany) were used to collect the fractions of dose released during the exhalation and inhalation phase of the breathing cycle. The nebulization process was stopped one minute after the occurrence of sputtering (the point at which the nebulizer stops continuous work). The budesonide deposited on the filters as well as the one remained in the chamber and T-piece were extracted with methanol/water solution (70:30) and quantified by high performance liquid chromatography (HPLC) as detailed previously (19). Three determinations were performed for each preparation.

*Aerodynamic diameter*

**Figure 2:**
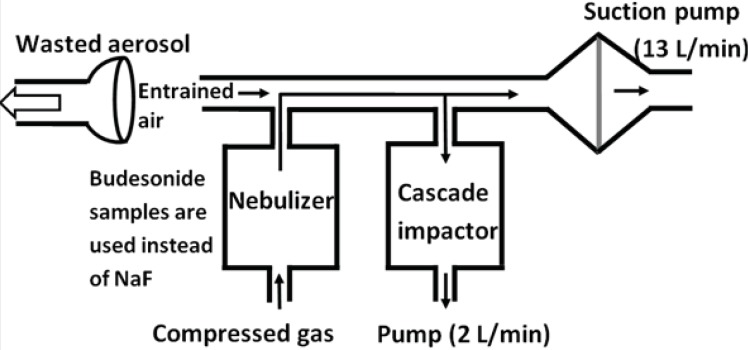
Schematic diagram aerodynamic particle size measurement

From the CEN methodology for the aerodynamic characterization of the emitted dose, stages of the Marple 298 x cascade impactor represent the cut off values of 50, 21.3, 14.8, 9.8, 6.0, 3.5, 1.55, 0.93 and 0.52 μm from top to the bottom, respectively. Amounts of budesonide ND and commercial suspension were determined in each stage. The airflow through the cascade impactor was set at a continuous flow of 2 L/min, while a further 13 L/min drawn from a second pump connected between the cascade impactor and the nebulizer (see Figure 2). Accordingly, the total airflow across the outlet of the nebulizer is 15 L/min. Budesonide deposited onto glass filters (Omega speciality instruments, USA) of each stage was extracted by methanol-water solutions and quantified using HPLC. The Copley inhaler testing data analysis software (CITDAS, Copley Scientific, UK) was used to calculate the mass median aerodynamic diameter (MMAD), fine particle fraction (FPF) (*i.e*. the percentage of particles with an aerodynamic diameter less than 5 μm), and geometric standard deviation (GSD). A student t-test was used to examine the differences between the studied groups.

*Physical stability*

In order to check the physical stability of budesonide nanodispersion, particle size of the prepared budesonide ND, kept in glass containers at 4 °C and at 25 °C, was monitored. The changes in the particle size may be considered as indication of physical instabilities.

## Results

*Solubility studies*

**Figure 3:**
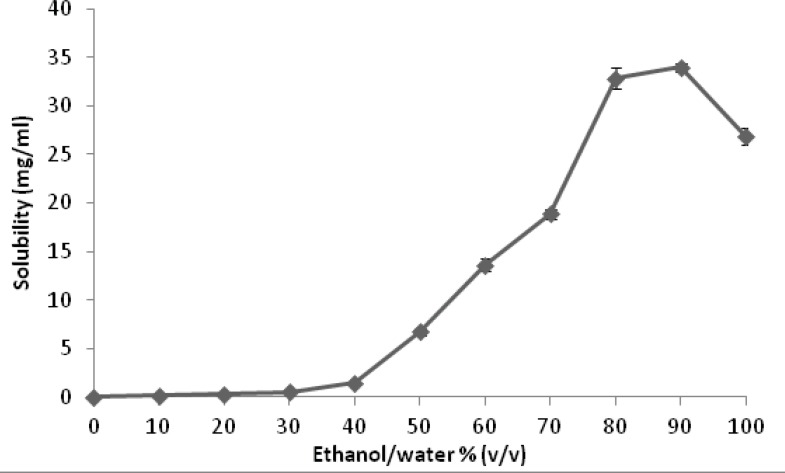
Solubility of budesonide in different ethanol-water combinations at 25 °C (n=3).

Figure 3 represents the results of solubility studies of budesonide in solvent (ethanol), antisolvent (water) and different ethanol-water combinations. Starting with low aqueous solubility (0.028 mg/mL), the solubility of budesonide was found to gradually increase by addition of ethanol until ethanol/water ratio of 40:60. Above this point, a marked increase in budesonide solubility was observed with further addition of ethanol until a mean (standard deviation, SD) of 34.00 (0.61) mg/mL at 90:10, ethanol: water, followed by a decrease to a value of 26.84 (0.29) mg/mL in pure ethanol.

*Microfluidic nanoprecipitation*

Budesonide nanodispersions (Z-Ave, 150 to 350 nm) with narrow size distribution (PDI, 0.10 to 0.22) were prepared using different microreactors under different processing conditions. As the Table 1 shows, the different processing conditions studied do not seem to be substantially affecting the size/polydispersity. Variation in flow rate of antisolvent was found to have a major effect on the drug particle size. The data also show that relatively smaller particles were generated with increasing drug concentration. In addition, it was observed that smaller particles may be generated using microreactors with smaller-sized internal diameters and/or with decreasing inlet angle.

*Particle size analysis of the tested preparations*

PCS analysis of the budesonide ND showed that the mean particle size was 160 nm and PDI was 0.15. Whereas, the mean particle size of the marketed suspension was 3802 nm and PDI was 0.2. This finding is in agreement with previous reports stating that the particle size of budesonide of the marketed suspension are 2-3 μm in diameter (20) or 4400 nm (9). 

*Budesonide particles morphology*

**Figure 4:**
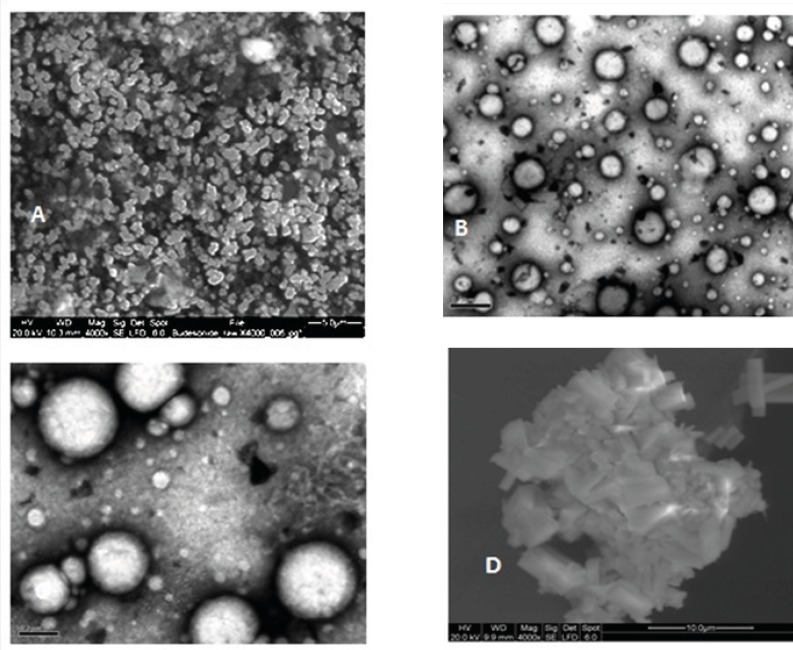
Morphology of different budesonide particles A. Unprocessed budesonide (SEM, bar = 5 μm), B and C. Dried nanodispersion (TEM, bar = 0.5 and 0.2 μm, respectively), D. Dried commercial suspension (SEM, bar = 10 μm).

Micrographs obtained using SEM and TEM, as complementary works to PCS, showed marked differences between budesonide particles. Unprocessed budesonide particles are irregular shaped crystals with diameter less than 5 μm (Figure 4A). After the nanoprecipitation process, budesonide particles appear to be spherical with smooth surfaces (Figures 4B and 4C). The observed diameter of dried budesonide ND was found to be smaller than the hydrodynamic diameter obtained by PCS analysis which is reported before (21). Budesonide particles from the commercial suspension are larger with sizes of 3-4 μm (Figure 4D). It is worth noticing that the agglomerations observed in the EM pictures might be due to the drying processes carried before EM.

*Aerosolization study*

*Particle size distribution*

**Figure 5:**
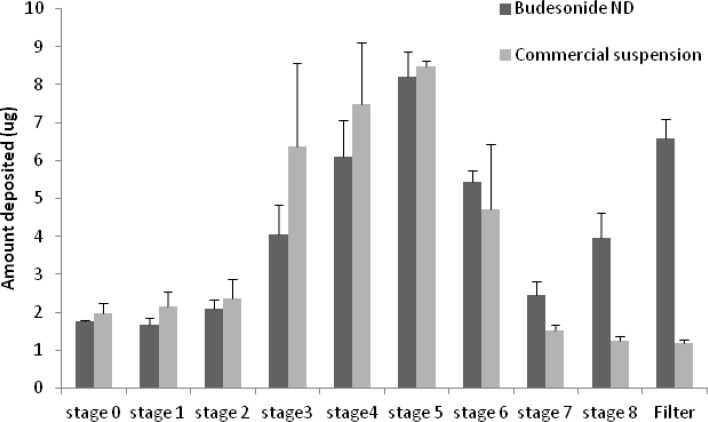
Deposition of budesonide (μg) on each stage of the cascade impactor using the CEN

Figure 5 displays the deposition pattern of budesonide ND and the commercial suspension as quantified from different stages of cascade impactor. The MMAD value in the microsuspension was significantly (p < 0.05) larger than MMAD of budesonide ND (see Table 2). Furthermore, the fine particle fraction (*i.e. *the fraction less than 5 μm) was significantly smaller (p < 0.05) in case of microsuspension compared with that of budesonide ND (Table 2). These data indicate better performance for the nanodispersion.

method for the budesonide ND and the marketed microsuspension (1 mg in 2 mL of budesonide as nominal dose) (n=3).

**Table 2:** The mean (SD) results obtained from cascade impactor for the marketed microsuspension and budesonide nanodispersion aerosolized using the Sidestream jet nebulizer (n=3).

**Preparation** **Parameter**	**Budesonide nanodispersion **	**Budesonide microsuspension**
FPF (%)	56.88 (3.37)	38.04 (7.82)
MMAD (μm)	3.91 (0.49)	6.22 (1.09)
GSD	2.86 (0.26)	2.20 (0.30)

*Aerosol output*

The results of the aerosol output studies (see Table 3) showed that the nebulisation time for budesonide ND is significantly (p < 0.05) shorter than that of the microsuspension. 

Overall, the comparative dose delivered to lung (estimated by multiplying the FPF and the % of dose retained on inhalation filter (20)) achieved by budesonide ND is 8.10 % of nominal dose compared with 5.68 % for the commercial microsuspension.

**Table 3:** The mean (SD) emitted dose of budesonide from the jet nebulizer (n=3).

	**Budesonide** **Nanodispersion **	**Budesonide** **Microsuspension **	
Inhalation Filter (percent of the nominal dose)	14.24 (2.51)		14.97 (4.3)
Exhalation Filter (percent of the nominal dose)	14.92 (3.21)		15.27 (4.48)
Chamber (percent of the nominal dose)	69.33 (5.08)		68.36 (8.95)
Connector (percent of the nominal dose)	1.49 (0.33)		1.39 (0.20)
Nebulization time (min)	12.30 (0.37)		14.85 (0.36)

*Physical stability*

**Figure 6:**
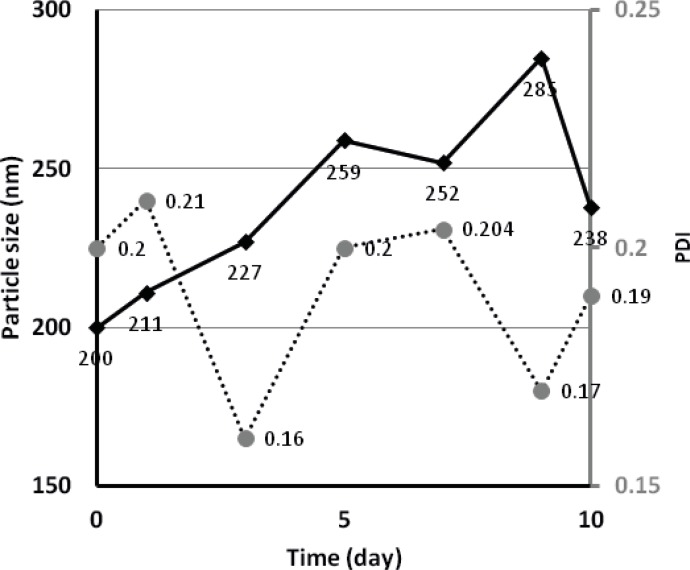
Change in particle size and polydispersity index (PDI) of budesonide nanodispersion stored at 4 °C (n=1).

For budesonide ND stored at room temperature, peaks of larger particles (5067 nm) and crystal sediments started to appear after 4-5 days of storage. However, samples stored at 4 °C, were found to have a particle size fluctuating between 200-300 nm over a period of 10 days (see Figure 6), indicating a more stable preparation. The measured zeta potential of budesonide ND was -7.93 and -2.00 when using water only and water containing polysorbate 80 as antisolvent, respectively.

## Discussion

Fluid flow in microchannels is laminar. Accordingly, the nano-sized particles are generated in the interface of the solvent and antisolvent through a diffusion procedure. The organic solvent containing the drug molecules compensates the depletion of budesonide from the diffusion layer due to nucleation and/or particle growth. This process involves supersaturation, formation of drug nuclei, and particle growth. The nuclei size start to increase and eventually the particles precipitate. Nevertheless, addition of surfactants or polymers has been reported to control the growth/precipitation rate (5, 22).

*Solubility studies*

This study employed ethanol as a solvent system since it is water-miscible, pharmaceutically acceptable and commonly used in inhalation products. The solubility experiments showed an increase in the solubility of budesonide by addition of ethanol to water. Nevertheless, the solubility decreases when the solvent contains 100% ethanol compared with a solvent system of ethanol/water (90:10). Similar solubility patterns have been observed for other drug molecules including lamotrigine, diazepam, and clonazepam in ethanol + water mixtures (23). The findings would provide guidance for the amounts of budesonide that could be precipitated using different ratios of solvent and antisolvent in the microfluidic reactors experiments.

*Microfluidic nanoprecipitation*

The results of the effect of different processing conditions on the size/polydispersity showed that the effects appear to be complex, as discussed previously using artificial neural networks (ANNs) (7). The effect of flow rate is most probably due to inducing higher supersaturation and drug nucleation at higher antisolvent flow rates when keeping solvent rate constant. This results in reducing the size of generated particles, as reported previously (7). Higher supersaturation and nucleation, made by increasing drug concentration makes the particles smaller, as indicated in this work. Also, as a result of better mixing process, smaller internal diameters make the particles smaller (24). Furthermore, budesonide particle size was found to decrease using microreactors with reducing inlet angle as reported before (7). 

*Aerosolization studies*

The results obtained from the PCS showed approximately 20-fold decrease in the particle size of the budesonide ND compared to the corresponding microsuspenison. It is already shown that decreasing the size to obtain nanoparticles, makes a considerable improvement in the rate of drug absorption (25). Therefore, a better *in-vivo *efficacy may be expected, provided an improved aerosolization performance is observed.

The aerodynamic size studies performed in this work, showed a better *in-vitro *behavior in the nanodispersion compared with the microsuspension. With the two preparations containing nearly equal amounts of surfactant and drug, it is hypothesized that the difference in budesonide particle size between the commercial microsuspension and budesonide ND is the primary and dominant factor for the difference in drug disposition between the two preparations. The smaller drug particles require reduced energy to be aerosolized and incorporated in aerosol droplets. Thus, smaller aerosol droplets are generated from the nebulizer. 

However, considering the details, some ultra small aerosol droplets may be observed (see filter in Figure 5), indicating a bimodal pattern in the size distribution of aerosol droplets. It is arguable that during the nebulization, few (or a single) nanoparticles get-together to form the small and ultra small particles (stage 8 and filter in Figure 5, respectively), while larger aerosol droplet contain more particles. Such aerosol droplets reported in Figure 5 may be exhaled before depositing in the respiratory airways (26). On the other hand, it is already reported that such particles may deposit in the alveolar region of lung by diffusion, an advantage to employ the nano-preparations (27, 28). Undoubtedly, generalizing our *in-vitro *findings will only be possible when *in-vivo *studies are performed too. Especially, when considering the fact that similar to many other corticosteroids, budesonide reaches the receptor by diffusing into cells. Therefore, faster dissolution in the airways is preferred which is obtained when having nano-sized particles (14).

The aerosol output results showed a slightly shorter nebulisation time for the nanodispersion. This is advantageous as lengthy treatment times may lead to poor compliance of the patient. Moreover, analysis of inhalation filters indicated that nearly equal amounts of budesonide were recovered from the commercial product and budesonide ND. Also, the amounts of budesonide recovered from exhalation filters and remaining in the nebulizer chambers were similar for both tested formulations.

In total, an approximately 1.4 fold increase in the dose delivered to the lung may be estimated for the nanodispersion compared with the commercial preparation. This is another indicator of better performance of nebulized nanodispersion *in-vitro*.

*Physical stability*

The bottom-up prepared nano-budesonide dispersion was prepared using polysorbate 80, as a stabilizer, similar to the commercial microsuspension. The stabilizing effect of polysorbate 80 comes from its ability to be adsorbed and cover the generated particle surfaces (sterical effect). The changes in the size observed in this work, might be from dissolution of drug nanoparticles (in case of decreasing particle size) or particle growth due to Ostwald ripening and/or occasionally agglomeration of the particles (29). The difference in zeta potential values may arise from adsorption of the non-ionic stabilizer on drug particles resulting in increase in the thickness of the diffuse double layer and hence a lower zeta potential (30).

## Conclusion

The capability of bottom-up prepared budesonide nanodispersions as alternatives to the corresponding microsuspension formulations for delivery to the lungs using nebulizers has been demonstrated. The overall results showed similar patterns for the *in-vitro *aerosol output studies. However, the aerodynamic droplet size and the nebulization time were improved when using the nanodispersion compared with the microsuspension indicating improved *in-vitro *performance for this formulation. 
